# Severe Acute Respiratory Syndrome Coronavirus 2 Infection among Returnees to Japan from Wuhan, China, 2020

**DOI:** 10.3201/eid2607.200994

**Published:** 2020-07

**Authors:** Yuzo Arima, Satoshi Kutsuna, Tomoe Shimada, Motoi Suzuki, Tadaki Suzuki, Yusuke Kobayashi, Yuuki Tsuchihashi, Haruna Nakamura, Kaoru Matsumoto, Asuka Takeda, Keisuke Kadokura, Tetsuro Sato, Yuichiro Yahata, Noriko Nakajima, Minoru Tobiume, Ikuyo Takayama, Tsutomu Kageyama, Shinji Saito, Naganori Nao, Tamano Matsui, Tomimasa Sunagawa, Hideki Hasegawa, Kayoko Hayakawa, Shinya Tsuzuki, Yusuke Asai, Tetsuya Suzuki, Satoshi Ide, Keiji Nakamura, Yuki Moriyama, Noriko Kinoshita, Yutaro Akiyama, Yusuke Miyazato, Hidetoshi Nomoto, Takato Nakamoto, Masayuki Ota, Sho Saito, Masahiro Ishikane, Shinichiro Morioka, Kei Yamamoto, Mugen Ujiie, Mari Terada, Haruhito Sugiyama, Norihiro Kokudo, Norio Ohmagari, Makoto Ohnishi, Takaji Wakita, the COVID-19 Response Team

**Affiliations:** National Institute of Infectious Diseases, Tokyo, Japan (Y. Arima, T. Shimada, M. Suzuki, T. Suzuki, Y. Kobayashi, Y. Tsuchihashi, H. Nakamura, K. Matsumoto, A. Takeda, K. Kadokura, T. Sato, Y. Yahata, N. Nakajima, M. Tobiume, I. Takayama, T. Kageyama, S. Saito, N. Nao, T. Matsui, T. Sunagawa, H. Hasegawa, M. Ohnishi, T. Wakita);; National Center for Global Health and Medicine, Tokyo (S. Kutsuna, K. Hayakawa, S. Tsuzuki, Y. Asai, T. Suzuki, S. Ide, K. Nakamura, Y. Moriyama, N. Kinoshita, Y. Akiyama, Y. Miyazato, H. Nomoto, T. Nakamoto, M. Ota, S. Saito, M. Ishikane, S. Morioka, K. Yamamoto, M. Ujiie, M. Terada, H. Sugiyama, N. Kokudo, N. Ohmagari)

**Keywords:** Asymptomatic infections, bias, pneumonia, prevalence, quarantine, COVID-19, 2019 novel coronavirus disease, coronavirus disease, SARS-CoV-2, severe acute respiratory syndrome coronavirus 2, viruses, respiratory infections, zoonoses

## Abstract

In early 2020, Japan repatriated 566 nationals from China. Universal laboratory testing and 14-day monitoring of returnees detected 12 cases of severe acute respiratory syndrome coronavirus 2 infection; initial screening results were negative for 5. Common outcomes were remaining asymptomatic (n = 4) and pneumonia (n = 6). Overall, screening performed poorly.

With the emergence of severe acute respiratory syndrome coronavirus 2 (SARS-CoV-2) in Wuhan, China, several countries, including Japan, repatriated their nationals (*1*–*3*). During January 29–31, 2020, a total of 566 Japanese nationals were repatriated via 3 chartered flights from Wuhan (206, 210, and 150 passengers). After passengers disembarked in Tokyo, Japan, quarantine officials assessed them for signs/symptoms (e.g., fever, respiratory illness) of coronavirus disease (COVID-19) (*4*). A total of 28 symptomatic passengers were transferred to select hospitals for isolation. The remaining 538 were transported to a designated hospital, where another 35 were found to be symptomatic and were hospitalized there or transferred to other hospitals, leaving 503 asymptomatic persons for observation in quarantine (Figure).

**Figure:**
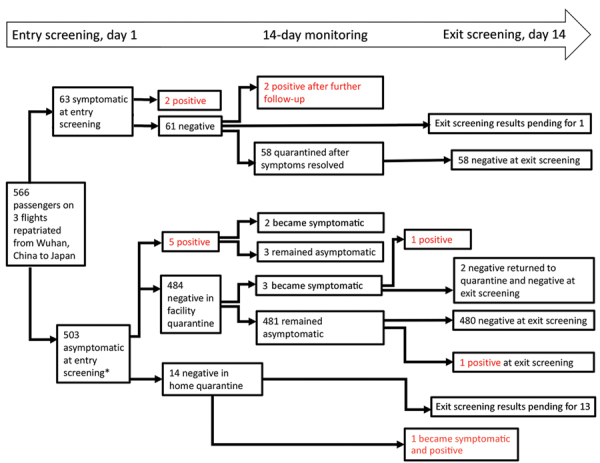
Results of testing 566 Japanese returnees from Wuhan, China, for severe acute respiratory syndrome coronavirus 2 by real-time reverse transcription PCR, January–February 2020. *Two persons were sampled on day 3, when they provided informed consent.

## The Study

We conducted day 1 entry screening by testing oropharyngeal swab samples collected from all 566 returnees at the hospitals to which they were initially transported for SARS-CoV-2 (*4*); all tests were based on the real-time reverse transcription PCR developed by the National Institute of Infectious Diseases (*5*). Hospitalized patients in isolation and asymptomatic returnees in quarantine were monitored daily for 14 days. If any signs/symptoms developed in a quarantined person, that person was transported to a designated hospital and oropharyngeal swab samples were collected for testing. We conducted exit screening for quarantined persons who remained illness-free by collecting oropharyngeal swab samples on day 14. The National Institute of Infectious Diseases Ethics Committee approved the study (registration no. 1096), and all 566 returnees who provided specimens gave written informed consent.

Among the 63 passengers who were symptomatic at entry screening, 2 (3.2%) were positive by PCR (Figure); test results were subsequently positive for 2 more. For 1 of these patients, pneumonia was diagnosed on day 1 and a sputum sample was positive on day 3; the other patient had fever and cough on day 1, pneumonia diagnosed on day 2, and a positive oropharyngeal swab sample on day 6. Excluding 1 patient who remained hospitalized for stroke, the remaining 58 patients were transferred to designated quarantine facilities after confirmation of good health and negative PCR results; all 58 remained asymptomatic after discharge, and PCR results were negative at exit screening.

For the 503 asymptomatic/subclinical passengers, entry-screening PCR results were positive for 5 (1.0%) (Figure); 3 remained asymptomatic, but mild signs/symptoms (fever, headache, sore throat) developed for 2 persons (1 on day 2, 1 on day 4). Of the remaining 498 persons with negative PCR results, 484 were quarantined at designated facilities and 14 at home. During quarantine, fever developed in 1 facility-quarantined and 1 home-quarantined person on day 10; both were confirmed positive by PCR, and pneumonia subsequently developed in both. The facility-quarantined case-patient was in a single room; no other person from this facility acquired COVID-19 or had a positive test result at exit screening. One person who remained asymptomatic had a positive test result at exit screening. Exit-screening results are pending for the patient hospitalized for stroke and the remaining 13 home-quarantined persons.

Among the 566 returnees, 12 cases of SARS-CoV-2 infection were detected; 540/541 facility-quarantined persons were confirmed negative by PCR performed on days 1 and 14 (197/197, 199/199, and 144/145 for the 3 flights). Entry screening detected 7 infections, for an infection point prevalence of 1.2%; infection period prevalence was 2.2% (12/552 returnees with complete follow-up). Despite universal testing, entry screening captured only 7/12 cases (58.3% sensitivity). Although screening symptomatic passengers (3.2%) was more efficient than screening all passengers (1.2%), screening only symptomatic passengers missed 5/7 prevalent infections at entry. Among symptomatic passengers, with 2 initially negative persons subsequently testing positive, entry-screening sensitivity was 2/4 (50%). Among asymptomatic passengers, with 3 initially negative persons subsequently testing positive, entry-screening sensitivity was 5/8 (62.5%).

## Conclusions

Testing all returnees—with follow-up for disease onset and course—enabled us to evaluate the spectrum of severity for SARS-CoV-2 infections (Table). From least to most severe, 4 patients experienced asymptomatic infection, 2 mild illness, and 6 pneumonia. Prospective monitoring proved essential because of the 7 prevalent infections at entry, 5 were asymptomatic, 1 mild, and 1 pneumonia. Even with potential underascertainment of asymptomatic cases because of a lack of serologic assessment (*6**,**7*) (i.e., interval-censoring during screening tests), it is noteworthy that 4/12 persons with infections were asymptomatic. Although numbers are small, severity seemed to be age dependent (Table). No infections were detected among the 100 persons <30 years of age; of the 2 infections detected among the 138 persons 30–39 years of age, both persons were asymptomatic. Although no person in this study died, only 1 was >69 years of age. Regarding sex, excluding 1 returnee for whom sex was unknown and 14 for whom exit-screening results are pending, of the remaining 551 returnees, 9 (1.8%) of the 506 male passengers (2 asymptomatic, 2 mild, 5 pneumonia) and 3 (6.7%) of the 45 female passengers (2 asymptomatic, 1 pneumonia) were infected.

**Table:** Distribution of severe acute respiratory syndrome coronoavirus 2 infections and clinical outcomes, by age group, among 566 Japanese returnees from Wuhan, China, January–February 2020

Age group, y	No. returnees*	No. (%) infected†	Outcome			
			No. asymptomatic	No. with mild illness	No. with pneumonia	No. deaths
<10	6	0	0	0	0	0
10–19	4	0	0	0	0	0
20–29	90	0	0	0	0	0
30–39	138	2 (1.4)	2	0	0	0
40–49	168	4 (2.4)	0	1	3	0
50–59	119	5 (4.2)	1	1	3	0
60–69	26	1 (3.8)	1	0	0	0
70–79	1	0	0	0	0	0

*Day-14 exit-screening test results pending for 14 persons.  †Testing by reverse transcription PCR.

Our findings have public health implications. As recently reported (*1*), we found that symptom-based screening performed poorly, missing asymptomatic and presymptomatic cases. Even with universal screening, nearly half of cases were missed. Because an asymptomatic case was detected at exit screening, limiting testing of quarantined persons to those with signs/symptoms would have missed such a case; with exit-screening results pending for 14 returnees, sensitivity could be lower. The poor sensitivity of single-point testing highlights the challenges of detecting SARS-CoV-2 infections.

The potentially long incubation period of COVID-19 was consistent with that recently reported (*8**,**9*) and contributed to the large proportion of missed cases. Active daily monitoring ensured that specific illness-onset times were captured, protected from the limitations associated with patient recall of symptom onset (*10*). Although exposure to SARS-CoV-2 occurred at some time before quarantine (i.e., left-censored), our setting enabled us to estimate the minimum incubation period for each incident symptomatic case by taking the return date as the exposure time. Determining the specific exposure time can be difficult and is conditional according to the definition of contact. Given such qualifications, a conservative minimum incubation period of 10 days obtained prospectively in a clean quarantine setting, without recall or assumptions regarding transmission modes, is noteworthy.

Testing and follow-up of all returnees provided valuable information about the spectrum of SARS-CoV-2 infection. Most reported data have been from medically attended patients, skewed toward symptomatic patients and more severe cases, limiting our knowledge of the clinical spectrum of infection (*6**,**11*). In our setting, we could remove the influence of patients’ health-seeking behaviors and clinicians’ diagnostic practices and found that 4/12 case-patients were asymptomatic. At the same time, of the 8 case-patients who experienced symptoms, pneumonia developed in 6. Our findings were also consistent with the reported age-dependent nature of COVID-19 (*2*,*12**–**14*); infection and clinical attack rates were lower among younger persons. Shedding light on the severity pyramid among those infected—not only among those who sought care—provides an evidence base for risk communication, healthcare planning, and public health response. Combined with reports suggesting transmissibility of SARS-CoV-2 from asymptomatic/subclinical case-patients (*1*,*10**,**15**,**16*), our findings suggest that controlling COVID-19 through the usual tools of syndrome-based surveillance and contact tracing alone may be difficult.

When confronted with an emerging pathogen, researchers can generate critical epidemiologic information by studying quarantine populations. As with the First Few X study (*7*), our design is protected from the usual biases of passively reported surveillance data. Aggregating high-quality data from these types of investigations can build a larger severity pyramid, enabling reliable estimation of various severity measures (e.g., symptomatic proportion of infected case-patients, case severity proportion among those who are symptomatic). We recommend using similar assessments to help elucidate the epidemiology of SARS-CoV-2 and inform public health response.
